# Assessing the Effects and Acceptance of Silver Diamine Fluoride Treatment in Early Childhood Caries

**DOI:** 10.7759/cureus.55767

**Published:** 2024-03-08

**Authors:** Nishi Anant, Niharika Rai, Sowmya NR, Pramila Amaltas, Mrunal Kalambe, Ashwarya Emmanuel

**Affiliations:** 1 Department of Pedodontics and Preventive Dentistry, Triveni Institute of Dental Sciences Hospital and Research Centre, Bilaspur, IND; 2 Department of Pedodontics and Preventive Dentistry, Dr. Rajesh Ramdasji Kambe Dental College and Hospital, Akola, IND

**Keywords:** preventive treatment, early childhood caries, caries arrest, anti-caries, silver diamine fluoride

## Abstract

Background: Early childhood caries (ECC) is a widespread dental problem that impacts children below the age of six years. Traditional restorative treatments like fillings are often challenging and invasive for young children. Silver diamine fluoride (SDF) offers a minimally invasive and cost-effective alternative for managing ECC. However, the effects, acceptance, and understanding of this treatment by parents remain crucial for its successful implementation.

Aim: To evaluate the impact and level of acceptance of SDF treatment in ECC.

Materials and Methods: Thirty-two children from an Anganwadi center aged between two to five years suffering from ECC were selected. A 38% SDF was applied for 3 minutes on the identified carious lesion, and the arrest percentage was checked after a recall period of three weeks and three months. The Likert scale was used for the questionnaire regarding parental feedback about the ease of application procedure, tooth discoloration, possible pain associated with the procedure, and the taste of SDF.

Results: In the present study, a total of 75 surfaces were subjected to the application of SDF. After a period of three weeks, it was observed that 64 of these surfaces had successfully undergone the process of caries arrest. This finding was deemed to be statistically significant, with a P value of 0.021. Furthermore, the remaining surfaces that had not achieved caries arrest were subsequently treated at three months. The results of this subsequent treatment were also found to be statistically significant, with a P value of 0.027. These findings highlight the efficacy of both caries treatment and caries arrest in relation to the utilization of SDF.

Conclusion: SDF was found to be minimally invasive, easy to administer, inexpensive, highly efficient, and effective treatment in arresting caries in the primary dentition of young children, particularly in patients lacking cooperation.

## Introduction

Dental caries remains a significant oral health challenge in numerous developing nations [1]. Prompt and effective treatment of dental caries is of utmost importance as untreated caries can lead to a decline in a child’s general health and quality of life [2]. The efficacy of silver diamine fluoride (SDF) as a means of preventing early childhood caries (ECC) is increasingly recognized. SDF has emerged as an anti-caries agent, capable of effectively halting the progression of dental caries, offering a potential solution to combat the widespread issue of untreated dental decay in young children [3,4]. The bactericidal properties of SDF are attributed to its free silver ions, which bind to bacterial surfaces and induce cell damage. Moreover, SDF has the ability to remineralize affected surfaces by facilitating the deposition and incorporation of fluoride ions. These actions make SDF a valuable tool in the prevention and treatment of ECC.

Utilizing SDF offers the advantage of deferring or averting dental intervention until a child is more cooperative, making it a non-surgical alternate for addressing caries in communities where surgical management is not feasible [5]. Furthermore, employing SDF to chemically arrest active caries eliminates the necessity for local anesthesia and electric or air turbine handpieces, ultimately enhancing the dental experience for children with caries.

The topical application of SDF has gained significant attention due to its effectiveness, affordability, and simplicity [6]. Systemic reviews have also indicated that SDF could play a crucial role in oral health promotion programs for children, aligning with the World Health Organization (WHO) Millennium Goals [6,7]. Nonetheless, further studies are required to validate the impact of SDF in a pediatric population.

SDF can be valuable in addressing the needs of demographics that experience high rates of dental caries. This includes children, bedridden patients, individuals with special healthcare needs, xerostomia, those undergoing post-radiation therapy and chemotherapy, and members of communities with very low socioeconomic conditions [3]. The pedodontist is typically the initial dental specialist to whom a child is brought for dental treatment. It is incumbent upon the pedodontist not only to evaluate the child's dental condition but also to prevent and intervene in potential issues. This underscores the importance of research and study within this particular specialty. 

Thus, the current study aims to evaluate the impact and level of acceptance of SDF treatment in ECC. 

## Materials and methods

A total of 32 children from an Anganwadi center were selected due to their limited access to dental care. The selected children were aged two to five years and lacked cooperative abilities. The study included children with at least one active carious lesion, free from pulpal pathology and tooth mobility and without any hereditary developmental disorders or known allergies to silver-containing dental materials. However, children under two years or over five years with tooth mobility, signs of pulpal pathology, hereditary developmental defects, or allergies to silver-containing materials were excluded from the study.

**Figure 1:**
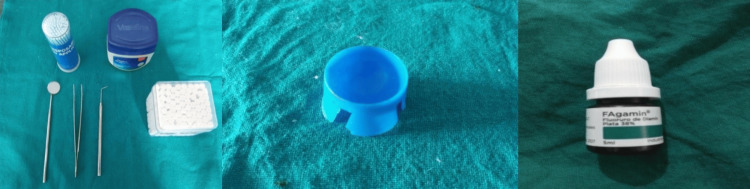
Armamentarium used in the study

Following a standard dental examination with a baseline assessment, the international caries detection and assessment system (ICDAS; score up to 3) was used to classify various stages of carious processes, extending from early visible changes in enamel to wide cavitation. To treat identified carious lesions in primary teeth, 38% SDF was used. A total of 70 teeth were treated with SDF application in 32 patients, out of which eight were anterior teeth and 62 were posterior teeth. Surface-wise examination of the carious lesions was done. Specific teeth were dried and isolated using gauze and cotton rolls with Vaseline applied over the mucosa and gingiva (Figure 1).

**Figure 2:**
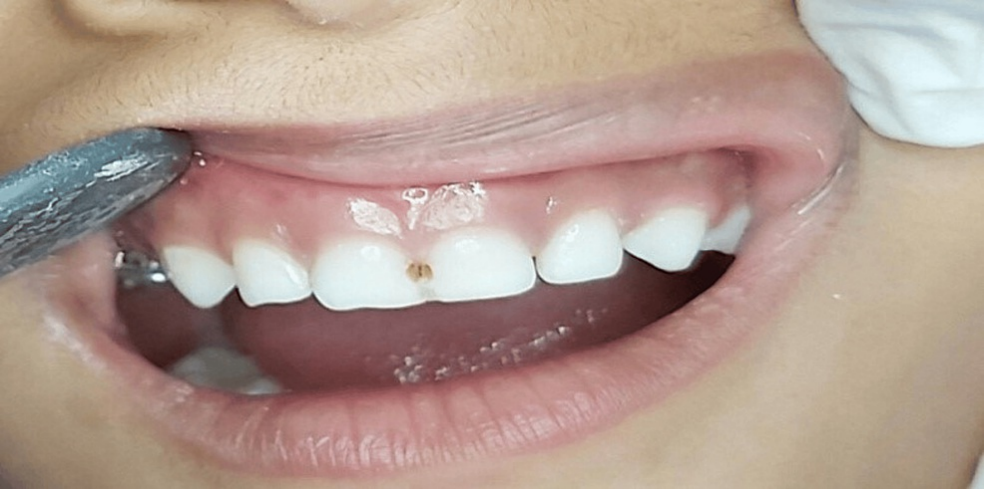
Pre-operative: Smooth surface caries seen with respect to 51, 61

**Figure 3:**
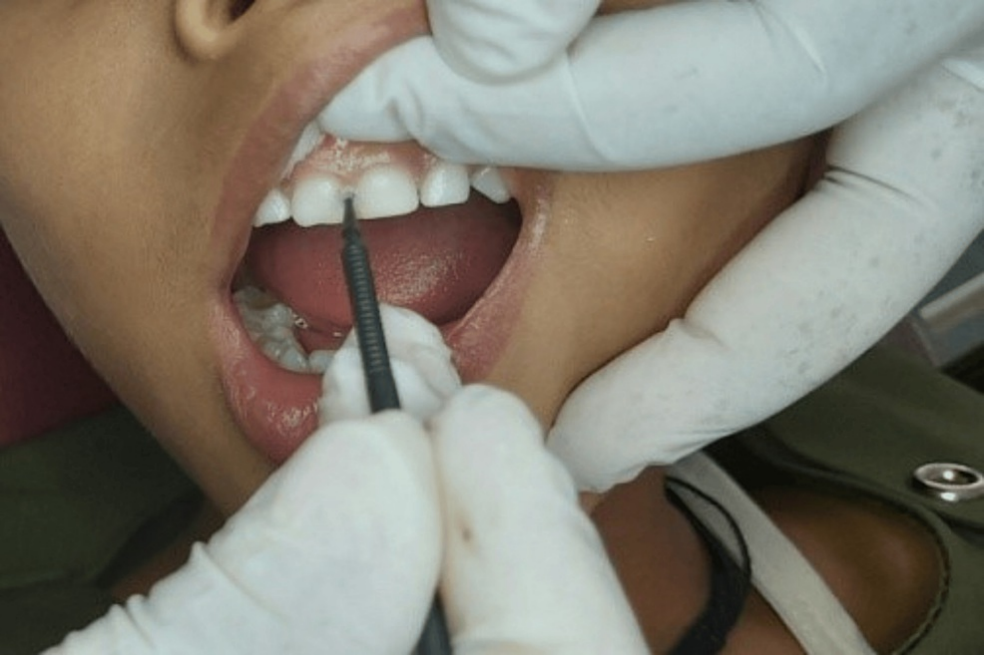
The teeth were isolated and SDF was applied over the carious lesion SDF: Silver diamine fluoride

**Figure 4:**
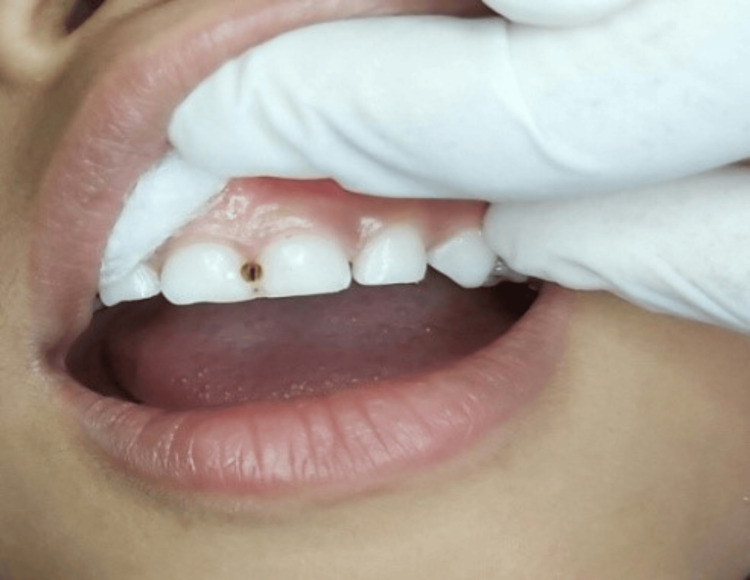
Post-operative: After SDF application, the carious lesion was found to be black with respect to 51, 61 SDF: Silver diamine fluoride

SDF drop was then dispensed in a plastic dispenser and applied to the lesion directly using a microbrush, allowing it to be imbibed for up to 3 min. Any excess amount of SDF was wiped up with the help of cotton. Parents were educated to ensure that their child must refrain from eating or drinking for one hour following the SDF application. The clinical pictures of pre-operation, SDF application, and post-operation are shown (Figures 2-4).

Lesions were reassessed at three weeks post-SDF application, and subsequently, at three months upon follow-up. If the carious lesion did not exhibit characteristics of being black and hard at either recall evaluation, indicating arrest, additional SDF application was done. The reapplication of SDF was documented at recall visits.

The endpoint was determined upon the identification of an arrested decay in the child. In order to evaluate the efficacy of arresting caries, clinical outcomes were assessed. Positive results were determined by the presence of dark, hard, and black lesions without pain or infection. Conversely, the treatment failure was indicated by the progression of the lesion manifesting as a yellow, soft lesion as well as the presence of pain or infection. Likert scale [8] was used for the questionnaire regarding the parental feedback about the ease of application procedure, tooth discoloration, possible pain associated with the procedure, and the taste of SDF. 

## Results

In this study, a total of 32 children were included, with 21 (65.6%) being males and 11 (34.4%) being females. The age-wise distribution of the study participants is as follows: three (9.4%) belonged to 2.5 years, five (15.6%) to 3 years, three (9.4%) to 3.5 years, eight (25%) to 4 years, six (18.8%) to 4.5 years, and seven (21.9%) to 5 years. Upon examination of caries on the surfaces of anterior teeth, single surface caries were present in four locations (12.5%), two surface caries in three locations (9.4%), and three surface caries in one location (3.1%). Similarly, a surface-wise assessment of caries in posterior teeth revealed that occlusal caries were present in 23 locations (71.9%), proximal caries in 20 locations (62.5%), buccal caries in nine locations (28.1%), and lingual caries in 10 locations (31.3%). A total of 75 surfaces were treated with SDF. Arrest percentage was calculated for treated surfaces. In this study, the relationship between the application time of SDF and the percentage of lesion arrests was assessed in children aged two to five years with active carious lesions at three-week follow-up. The arrest percentage for 120 seconds was 99.08%, for 30 seconds was 100%, for 45 seconds was 100%, for 60 seconds was 99.88%, for 75 seconds was 100%, for 90 seconds was 99.95%, and for 180 seconds was 99.72%. No statistically significant correlation was found between the SDF application time and arrest percentage (P value = 0.255) (Table 1).

**Table 1:** SDF application time and lesion arrest percentage at three-week follow-up SDF: Silver diamine fluoride

Time (s)	Surface treated (n)	Surface arrested (n)	Arrested percentage (%)
120	27	23	99.08
30	5	5	100
45	4	4	100
60	8	6	99.88
75	4	4	100
90	6	5	99.95
180	21	17	99.72

SDF demonstrated the ability to arrest caries in children diagnosed with ECC at an effective percentage rate. Following a single SDF application, out of 75 treated surfaces, 64 surfaces (92.96%) were observed to be arrested at the three-week follow-up, a statistically significant outcome (P value = 0.021). Furthermore, 11 surfaces with unarrested lesions were treated during the three-week follow-up, and these lesions were subsequently observed to be arrested at the three-month follow-up, establishing a statistically significant relation between caries treatment and caries arrest, with a P value of 0.027 (Table 2).

**Table 2:** Caries status of tooth surfaces before and after SDF application upon follow-up at three weeks and three months The level of significance is p<0.05. SDF: Silver diamine fluoride

At recall visits	Surfaces treated (n)	Surfaces arrested (n)	Arrest percentage (%)	P value
At three weeks	75 surfaces	64 surfaces	92.96	0.021^*^
At three months	11 surfaces (reapplication of SDF)	11 surfaces	100	0.027^*^

The median responses to various questions from parents were collected, revealing that the majority reported the SDF application procedure as painless for their child, with a "strongly agree" rating. Similarly, the acceptability of its taste also received a "strongly agree" rating. However, in response to inquiries about parental comfort level with discoloration following SDF application, the median response indicated "agree" (Table 3).

**Table 3:** Responses to different questions by parents SDF: Silver diamine fluoride

Question	Responses					Median response
	Strongly disagree (1) n(%)	Disagree (2) n(%)	Neutral (3) n(%)	Agree (4) n(%)	Strongly agree (5) n(%)	
Did you find the SDF application an easy procedure?	0 (0)	0 (0)	2 (6.3)	9 (28.1)	21 (65.6)	Strongly agree
Are you comfortable with the discoloration of the cavities after SDF application?	0 (0)	3 (9.4)	6 (18.8)	8 (25)	15 (46.9)	Agree
Was the SDF application procedure painless for your child?	0 (0)	0 (0)	1 (3.1)	6 (18.8)	25 (78.1)	Strongly agree
Was the taste of SDF acceptable to your child?	0 (0)	0 (0)	4 (12.5)	8 (25)	20 (62.5)	Strongly agree

## Discussion

ECC is a widespread public issue that results in significant pain and infection in many children, impacting physical development, diminishing learning abilities, and increasing future treatment expenses [9,10]. This dental disease affecting young children is multifactorial, infectious, and transmissible, particularly among vulnerable and low-income populations [11,12].

The effectiveness of SDF stems from the mixture of silver nitrate and fluoride. It operates through various mechanisms in preventing or halting dental caries. Firstly, it exhibits antibacterial action against cariogenic bacteria. Secondly, it promotes remineralization and inhibits demineralization. Lastly, it reduces the dentine collagen matrix by inhibiting collagenase.

SDF on reacting with hydroxyapatite crystals, results in the formation of silver phosphate and calcium fluoride. The calcium fluoride serves as a reservoir of fluoride ions, while the phosphate ions aid in remineralization. The silver ions within SDF infiltrate deep into carious lesions and exert their influence by reaching depths of up to 2 mm into a deep carious lesion. Black staining of the carious lesions is due to the presence of silver compounds like silver oxide and silver phosphate [13].

Several advantages of SDF are that it is simple to use, fast, non-invasive, painless, and effective procedure, with no need for local anesthesia. Further, there is less requirement for expensive equipment, and more importantly, it arrests and prevents the progression of dental caries. It can also be used in community dental set-ups with limited resources.

One of the inherent limitations of using SDF to arrest caries is the discoloration of the lesion that occurs following SDF application, as it causes the affected area to turn black. Despite this, SDF effectively halts the progression of caries by creating a tough, black, and impermeable layer on the tooth surface that is highly resistant to caries. Concerns have been raised regarding the potential toxicity of SDF due to the high fluoride concentration, approximately 44,800 ppm [14]. It is noteworthy that the amount of SDF typically applied per patient falls within a range of 0.17 mg to 0.36 mg, remaining well below the no observed adverse effect limit [15].

SDF treatment has shown 66% effectiveness in arresting caries in children. To further enhance its efficacy, it is now being combined with the atraumatic restorative technique (ART) in a method known as the silver-modified atraumatic restorative technique (SMART). In this technique, SDF is applied to the carious dentin after selectively removing the soft dentin. Subsequent visits involve the restoration or sealing of the treated area with glass ionomer cement, which not only restores the tooth's anatomy but also masks the discoloration caused by the SDF treatment. This approach provides a more aesthetically pleasing result [16,17]. Furthermore, the SMART technique is particularly useful for highly anxious children, serving as an interim alternative to traditional restorative techniques [18].

Different concentrations of SDF are commercially available, but 38% SDF is commonly used due to its effectiveness in arresting dental caries, particularly in primary teeth [19]. A study conducted by Yee R et al. [20] and Fung MH et al. [19] compared the efficacy of different SDF concentrations, specifically 12% and 38% SDF. Both studies concluded that 38% SDF was more effective in arresting caries than 12% SDF. In this study, we also used the 38% concentration of SDF to arrest caries in line with the findings of the previous studies.

In this study, we observed that SDF effectively arrested caries in children with ECC. After a single application of SDF, we found that out of 75 surfaces, 64 surfaces were arrested at the three-week follow-up. Only 11 surfaces required reapplication of SDF, and they were successfully arrested at the three-month follow-up. With the second application of SDF, nearly 100% of the caries were arrested.

This is in accordance with the study conducted by Clemens J et al. [21], wherein the effectiveness of SDF in arresting carious lesions was evaluated. After three weekly and three monthly intervals, a statistically significant difference in caries arrest percentage (98%) was reported post three months of SDF application. Similarly, Llodra et al. [22] found that the SDF application resulted in 97% of caries arrest at the six-month mark. Ruff et al. [23] observed an 80% caries prevention rate and concluded that SDF can be used in arresting and preventing caries in school-based oral health programs.

In terms of parental acceptability regarding the treatment, the majority of parents found the SDF procedure easy to carry out, comfortable, painless, and with acceptable taste for their child. This aligns with a study conducted by Cernigliaro D et al. [24], where caregiver acceptance of SDF treatment was found to be high.

SDF is known for its ability to permanently stain surfaces it comes into contact with, such as counters and clothing. One major concern with SDF application is the blackish discoloration it can cause on teeth, as well as accidental pigmentation of the skin and mucosal areas. A study by Patel J et al. [25] found that this black discoloration occurs due to the formation of a layer of silver phosphate on the carious dentin, along with the formation of silver sulfide precipitates. To address this issue, Nguyen V et al. [7] reported that applying potassium iodide (KI) after SDF application can help reduce the darkness caused by SDF.

The cost of managing severe ECC is disproportionately high, especially when advanced pharmacological behavior guidance modalities (sedation or general anesthesia (GA)) are needed. However, the use of SDF helps alleviate the economic burden and presents a definite advantage in treating ECC in difficult circumstances. Despite the fact that SDF may cause tooth discoloration, the benefits outweigh the drawbacks, especially in cases where access to dental care is a challenge.

Therefore, SDF has given fresh impetus to pediatric dentistry and can be an innovative dental material of the century. During the ongoing pandemic spread of COVID-19, it has proved to be a boon in the field of dentistry as SDF application is a non-aerosol generating procedure that does not cause viral transmission.

The study has certain limitations that warrant consideration. Firstly, the study's sample size was relatively small, and the follow-up time was limited. Future studies with larger sample sizes and longer follow-up periods are necessary to better understand the biological effects of SDF treatment. Secondly, the study was conducted within a specific Anganwadi center and focused on healthy young children aged two to five years. Therefore, the results may not be generalizable to other demographic groups, such as special needs children or older children. Additionally, parental acceptance of SDF treatment may vary across different population groups, which could affect the applicability of the study findings. Finally, while the study clinically evaluated the percentage of caries arrests, it is essential to acknowledge the need for further validation through histopathological and microbiological assessments to enhance the robustness of the results.

## Conclusions

In our study SDF was found to be minimally invasive, easy to administer, inexpensive, highly efficient and effective treatment in arresting caries in primary dentition of young children particularly in patients lacking cooperation. Parents found the SDF procedure, comfortable, painless and with acceptable taste for their children. Drawback of staining the teeth black was well accepted by parents as the benefits outweighed its adverse effect. However further studies are recommended with larger sample size in order to authenticate the findings of this study.
